# Clarifying Terminology in Chronic Cough: The Evolving Role of Cough Hypersensitivity Syndrome

**DOI:** 10.1111/crj.70205

**Published:** 2026-06-19

**Authors:** Zihang Wang, Yaxing Zhou, Yingchun Ren, Jiaying Yuan, Tongyangzi Zhang, Shengyuan Wang, Wanzhen Li, Li Yu, Xianghuai Xu

**Affiliations:** ^1^ School of Medicine Dalian Medical University Dalian Liaoning China; ^2^ Department of Pulmonary and Critical Care Medicine, Tongji Hospital, School of Medicine Tongji University Shanghai China

**Keywords:** chronic cough, cough hypersensitivity syndrome, disease management, phenotype, terminology as topic

## Abstract

Chronic cough is defined as a cough lasting no less than 8 weeks, and 10%–42% of patients with chronic cough retain their cough after comprehensive examination and treatment. The terms idiopathic chronic cough (ICC), unexplained chronic cough (UCC), and refractory chronic cough (RCC) are frequently used to describe this clinical condition. However, these terms are often used interchangeably in articles, leading to heterogeneity in the populations included in the studies and compromising the value of the findings in guiding clinical practice. This article aims to clarify the current terminological confusion by reviewing the definitions and relationships among ICC, UCC, and RCC in the literature, and to discuss the emerging role of cough hypersensitivity syndrome (CHS) as an important pathophysiological framework for understanding chronic cough, with a focus on its clinical value, current challenges, and future perspectives.

## Graphical abstract

This review clarifies the definitions and relationships among ICC, UCC, and RCC, and introduces cough hypersensitivity syndrome (CHS) as an important pathophysiological framework for understanding chronic cough. Within this framework, CHS provides a mechanism‐based perspective that may complement existing terminology, guide future research, and improve clinical communication and disease management.

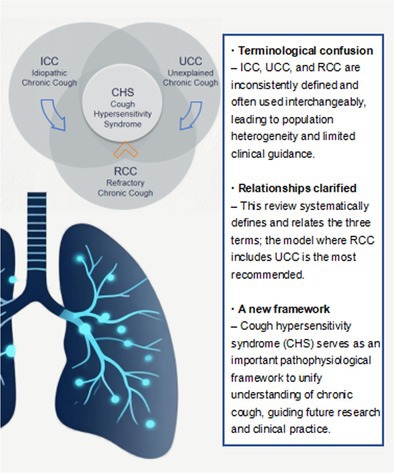

**Abbreviations**
CHS: cough hypersensitivity syndrome
COPD: chronic obstructive pulmonary disease
CVA: cough variant asthma
GERC: gastroesophageal reflux‐induced chronic cough
ICC: idiopathic chronic cough
LCQ: Leicester Cough Questionnaire
MII‐pH: multichannel intraluminal impedance‐pH monitoring
PROMs: patient‐reported outcome measures
RCC: refractory chronic cough
SSD: somatic symptom disorder
UACS: upper airway cough syndrome
UCC: unexplained chronic cough
URCC: unexplained or refractory chronic cough.

## 1 Introduction

Cough is an important physiological defense reflex of the respiratory system with the role of preventing the inhalation of foreign bodies and clearing airway secretions [1, 2]. Chronic cough is a cough that lasts longer than 8 weeks without abnormalities on chest imaging; it accounts for about 10% of the overall population and more than one‐third of respiratory clinic visits [3, 4]. Common etiologies of chronic cough include cough variant asthma (CVA), gastroesophageal reflux‐induced chronic cough (GERC), and upper airway cough syndrome (UACS) [5]. In clinical practice, 10%–42% of patients with chronic cough are still unable to identify the etiology after comprehensive examination or have no significant relief of cough after conventional treatment [6]. Terms such as unexplained chronic cough (UCC) and refractory chronic cough (RCC) are commonly used to describe these clinical conditions [7]. Disease management for patients with UCC/RCC poses a significant challenge in clinical practice. Patients who suffer from a prolonged and intractable cough may be at risk of physical complications, such as urinary incontinence, cough syncope, and dysphonia, as well as psychiatric disorders, including anxiety, depression, and somatic disorders, which can lead to significant decline in the patient's quality of life [8, 9, 10].

However, there is still no internationally accepted definition for such conditions. Terminology as Topic is therefore highly relevant, as the terms used to describe patients with UCC/RCC vary widely from study to study. So does the extent to which cases are evaluated for etiology. The description above demonstrates that the classification of UCC/RCC varies greatly across studies, which has a direct impact on the accuracy and effectiveness of research in guiding clinical practice. Therefore, deciding on a uniform terminology and definition is important and needs to be addressed urgently [7, 11, 12, 13]. This article reviews the origin and evolution of terms such as UCC/RCC, summarizes the definitions and relationships of UCC/RCC in published articles, and discusses the emerging role of cough hypersensitivity syndrome (CHS) as an important pathophysiological framework for understanding chronic cough, while considering UCC/RCC as potential clinical phenotypes within this framework.

## 2 Evolution of Commonly Used Terminology

In 1981, Irwin et al. used the term “chronic persistent cough” to describe cases that (1) eluded the diagnostic and therapeutic maneuvers of at least one referring physician, and (2) remained persistently troublesome to the patient for at least 3 weeks; the authors proposed an innovative diagnostic scheme based on the nerve pathways conducting the cough reflex [14]. They found that using the above diagnostic protocols, the etiology of chronic persistent cough was almost always determined, and etiology‐specific treatment was almost always successful [15, 16]. Therefore, the American College of Physicians recommended this step‐by‐step diagnostic approach in its guidelines for cough management published in 1998 [17]. The diagnostic protocol proposed by Irwin et al. has been valuable in guiding an etiologic diagnosis in patients with chronic cough. It represents a breakthrough in modern clinical medicine for the treatment of chronic cough.

Subsequently, the popularization of the diagnostic protocols for chronic cough may have led to differences in referral patterns. Several studies have found that, despite experiencing adequate diagnosis and experimental treatments based on anatomical diagnostic methods, the etiology of the cough is still unknown in some patients, and specific treatments to clarify the etiology of the cough are not always successful [18, 19]. Since then, different terminology has been generated for both the etiology and treatment of chronic cough. The terms used to describe the etiology of chronic cough include “idiopathic chronic cough” (ICC) and UCC. When referring to treatment outcomes, the term RCC is used. The terms ICC, UCC, and RCC describe a nonresponsive condition in response to etiologic investigations and therapeutic efficacy. New terms such as “cough hypersensitivity syndrome” (CHS) have appeared in recent articles. This new term is proposed to address some of the limitations of the UCC/RCC terms and to better describe the underlying pathophysiology of chronic cough [20, 21]. Different terms reflect different emphases, but they are often used interchangeably in articles, leading to a certain degree of confusion [7]. Chronic cough is a significant clinical issue for both patients and clinicians, but it is further complicated by imprecise terminology. We summarized the definitions, focus areas, and interrelationships of the ICC, UCC, RCC, and CHS (Table 1).

**TABLE 1:** Definitions, focus areas, and relationships of the ICC, UCC, RCC, and CHS.

Term	Definition	Primary focus	Relationship to other terms
ICC	Chronic cough with no identifiable cause after thorough investigation	Etiology; unknown cause	Largely replaced by UCC; considered a true clinical entity in some patients
UCC	Chronic cough persisting > 8 weeks despite investigation and therapeutic trials per guidelines	Etiology; unexplained	May overlap with RCC; preferred over ICC
RCC	Chronic cough unresponsive to guideline‐based investigation and treatment	Treatment response	Broad definition includes UCC; narrow definition excludes UCC
CHS	Clinical syndrome characterized by cough triggered by low‐level thermal, mechanical, or chemical stimuli	Pathophysiology; neural hypersensitivity	Encompasses UCC/RCC as phenotypes; proposed as unifying diagnosis

## 3 UCC Is the Preferred Term for Chronic Cough of Unknown Etiology

### 3.1 ICC Is a Real Clinical Entity

The anatomical diagnostic protocol established by Irwin et al. was designed to identify common etiologies of chronic cough such as CVA, GERC, and UACS [14]. In specialist cough clinics, some patients are still unable to identify the cause of their cough even after a thorough examination and assessment. The term ICC has been used to describe this clinical condition [22]. “Idiopathic” comes from the Greek and is defined in the Oxford Dictionary as a disease entity that is not caused by any disease. Whether idiopathic cough exists, and whether it is a distinct clinical entity, has been hotly debated during the period 1990–2010 [23, 24].

Whether ICC represents a true disease entity remains controversial. Some authors have suggested that ICC may reflect diagnostic uncertainty rather than a distinct clinical condition. In clinical practice, some cases with an underlying etiology may be misdiagnosed as ICC owing to differences in the degree of evaluation of intrapulmonary or extrapulmonary causes, the dosage of empirical treatments, and the duration of therapy [25, 26, 27]. For instance, the diagnosis of eosinophilic bronchitis requires a sputum eosinophil count. Clinics lacking induced sputum testing are at risk of misdiagnosing these patients with ICC [28, 29]. GERC is also a challenging issue in clinical practice [30]. Patients with nonacid reflux, who do not exhibit typical reflux symptoms, may be easily misdiagnosed, and the dosage and duration of anti‐reflux medication may alter the treatment effect; some patients even need to undergo anti‐reflux surgery to alleviate reflux‐induced cough [31, 32, 33, 34]. All of these factors can contribute to misdiagnosis.

However, several studies have suggested that a subset of patients may have true idiopathic cough despite comprehensive diagnostic evaluation [22]. These patients are truly free of any underlying cause of the cough, rather than having undergone an inadequate diagnostic assessment. A series of studies have found that patients with idiopathic cough have a specific phenotype. These patients are predominantly middle‐aged or postmenopausal women, with viral upper respiratory infections often serving as the initiating event. Additionally, they exhibit a persistent dry cough and a hypersensitive cough reflex [6, 22]. As the mechanisms of the cough reflex have been subjected to extensive investigation, it has been demonstrated that peripheral and central sensitization of the cough reflex pathway represents the primary pathophysiological mechanism of ICC [35]. The favorable results achieved by drugs targeting the cough reflex pathway in patients with ICC provide additional support for the hypothesis [36, 37, 38]. Consequently, although some patients are susceptible to misdiagnosis due to an inadequate evaluation, idiopathic cough without any underlying etiology is indeed a real clinical entity. Modulating the sensitivity of the cough reflex represents the most promising therapeutic approach for patients with ICC.

### 3.2 The Term ICC Was Replaced by the Term UCC

Although both ICC and UCC are etiology‐specific terms, meaning that the etiology is unknown after a patient has been fully evaluated, there are some differences in the use of the terms ICC and UCC. ICC is indicative of a single clinical entity, namely, that the case is truly free of any associated underlying etiology [24]. In contrast, UCC emphasizes that there is no clear etiology but does not completely exclude the possibility that the patient may have an associated etiology for their cough that has not yet been detected with current medical technology. Accordingly, the 2006 ACCP guidelines advocate using the term UCC instead of ICC, which implies that patients who have not yet received a definitive etiologic diagnosis may have an underlying etiology that is currently undetected [39, 40]. UCC was clearly defined in the 2016 guidelines as a cough that persists longer than 8 weeks and remains unexplained after investigation and supervised therapeutic trial(s) conducted according to published best practice guidelines. The author further noted that it can occur under three different circumstances: (1) chronic cough with no diagnosable cause (UCC), (2) explained but RCC, and (3) unexplained and RCC. Nevertheless, the authors also indicate that it is uncertain whether these distinctions are beneficial or essential [41]. The term UCC has largely replaced the use of ICC in subsequent publications.

## 4 Terminology for the Treatment Effect: RCC

RCC is employed to describe the effect of treatment, particularly in instances where the patient exhibits a lack of response to the administered therapies, and often has two different definitions in articles [7]. RCC is narrowly defined as a case with an etiology that causes chronic cough, such as GERC or CVA, but the cough remains unresolved despite adequate treatment for the etiology [42, 43]. For example, our group has proposed a definition of refractory GERC: a condition of chronic cough with objective evidence of abnormal reflux as demonstrated by multichannel intraluminal impedance‐pH monitoring (MII‐pH), which is resistant to 8 weeks of standard medical anti‐reflux treatment but responsive to the subsequent intensified anti‐reflux therapy [44, 45].

More widely accepted and recommended by the guidelines is the broad definition of RCC: a type of chronic cough that is persistent despite any investigation and treatment according to published practice guidelines [3, 11, 27, 46]. In the 2021 Chinese guidelines for the diagnosis and treatment of cough, RCC is defined as a cough of > 8 weeks' duration that is of unknown cause despite recommended standardized investigations and treatments, or a cough that is persistent despite specific treatments for known etiologies or empirical treatments for common etiologies [47]. Accordingly, the broad definition of RCC encompasses both refractory cough with a clear etiology and chronic cough with an unknown etiology.

Although RCC was originally defined according to treatment response rather than pathophysiology, accumulating evidence suggests that many patients with RCC share common clinical characteristics and underlying mechanisms, particularly cough hypersensitivity and neural dysregulation [38]. In this regard, RCC may be viewed as more than simply a treatment outcome and may represent a clinically meaningful disease phenotype. However, given the heterogeneity of RCC and the likelihood of multiple underlying mechanisms, current evidence is insufficient to support RCC as a single disease entity. Further studies are needed to determine whether specific biological endotypes can be identified within this population.

## 5 The Relationship Between UCC and RCC

The relationship between UCC and RCC is further complicated by the lack of a clear and harmonized definition of UCC/RCC. A review of the descriptions of UCC/RCC terms reveals that they can be summarized in three ways.

### 5.1 UCC and RCC Are Independent of Each Other

In this context, RCC refers to a patient who has an identified etiology for their chronic cough, but the specific treatment for that etiology is ineffective. The narrow sense of RCC indicates that the patient has a chronic cough‐related etiology, which distinguishes it from UCC. Such relationships are frequently observed in articles that compare the clinical characteristics of patients with UCC and RCC. The results of related studies indicate that there is no significant difference between patients with UCC and RCC in terms of demographics and cough characteristics [43, 48, 49, 50]. Consequently, further studies may be conducted to ascertain whether these terms correspond to distinct patient populations [49].

### 5.2 RCC Incorporates UCC

In this context, RCC refers to a cough that persists despite a series of diagnostic tests and treatments, which emphasizes that patients do not respond well to treatment [46]. Patients presenting with an UCC often demonstrate a lack of responsiveness to conventional cough suppressant therapy. Consequently, RCC encompasses UCC [11]. This type of relationship is by far the most accepted and clinically instructive. In the 2021 Chinese guideline, it is recommended that RCC be categorized into two distinct subtypes: RCC with a clear etiology and RCC with an unknown etiology. This classification guides the implementation of different treatment strategies for each category [47]. RCC with a clear etiology should first be treated with all etiology‐specific therapeutic measures to relieve the cough. For instance, surgery should be considered for patients with refractory GERC due to esophageal hiatal hernia [34]. Second, it is important to consider other factors that may influence the efficacy of treatment, such as patient compliance and the presence of cough‐related exposures in the living environment [51]. Following the above evaluation and intervention, some patients may experience improvement in cough symptoms. Patients who do not demonstrate significant improvement are treated in a manner analogous to those with RCC of unknown etiology. This entails a comprehensive evaluation and the subsequent selection of drugs targeting the pathways of the cough reflex for further treatment [38].

### 5.3 RCC Is Equivalent to UCC

RCC is defined broadly to include RCC of unknown etiology and RCC of clear etiology. Those who propose that UCC is equivalent to RCC are also classifying RCC with a clear etiology as UCC [12, 41, 52]. One potential explanation is that the cough persists despite treatment for the etiology, which suggests that the etiology may not be the real cause of the cough. This indicates that the underlying cause of the cough remains unknown. Therefore, RCC with a clear etiology but poorly treated against the etiology can be classified as UCC. In this instance, RCC is equivalent to UCC. This is the reason why the article refers to patients with cough of unidentified etiology and those who do not experience significant relief of their cough with treatment as having UCC/RCC, without distinguishing between them. The French guidelines for the management of chronic cough, published in 2023, utilize the term URCC (unexplained or refractory chronic cough) to define the relevant population [52]. Nevertheless, the unsatisfactory results of treatment for a known etiology suggest that this etiology may not be the underlying cause of the chronic cough. The matter remains a source of contention. The equivalence of RCC to UCC is not widely accepted or used.

## 6 CHS as an Important Framework for Understanding Chronic Cough

In recent years, chronic cough has been increasingly recognized as a distinct clinical entity, caused by sensitization of the neural pathways of the cough reflex [36, 53]. The concept of CHS was first proposed by the European Respiratory Society in 2014. CHS is defined as a clinical syndrome characterized by a cough caused by low‐level thermal, mechanical, and chemical exposures [20]. CHS has been proposed as an important framework for understanding chronic cough from a pathophysiological perspective. Within this framework, different etiologies, such as GERC, CVA, and UCC, may share common features of cough hypersensitivity and can be viewed as different clinical presentations associated with this mechanism. Several authors have suggested that the CHS concept may complement existing terminology and contribute to a better understanding of chronic cough. The following sections summarize the main advantages of this terminology [7, 54, 55].

### 6.1 Advantages of the CHS Terminology

1. CHS more accurately reflects the clinical characteristics described by patients, rendering it a highly clinically useful term. In clinical practice, patients with UCC/RCC often complain of a range of unusual sensations in the larynx, including tickling, irritation, dryness of the throat, soreness of the throat, and mucus in the throat. Additionally, they frequently experience strong coughing impulses in response to low‐level physiologic, mechanical, and chemical stimuli, such as changes in ambient temperature, deep breathing, laughing, and talking on the phone [21, 48, 56]. The term CHS is consistent with the patient's self‐described characteristics and facilitates the physician's clinical recognition of the patient's symptoms.
2. Furthermore, CHS is regarded as an important underlying pathophysiological mechanism of UCC/RCC, namely, hypersensitivity of the cough reflex [55]. The cough reflex pathway is primarily sensitized as a result of increased sensitivity of peripheral and central nerves that regulate cough [36, 57]. There is a degree of variability in the sensitivity of the cough reflex among individuals. This may also explain why some diseases (e.g., viral infections, and GERD) cause only a transient, self‐limiting cough in the majority of people, whereas in some patients, they can cause a persistent cough. This suggests that the disease causing the cough serves only as a trigger and that sensitization of the individual cough reflex is considered an important contributor to the persistence of chronic cough [20].
3. Targeting cough hypersensitivity represents an important therapeutic strategy for many patients with UCC/RCC [38, 58]. Patients with UCC/RCC often exhibit a poor response to conventional cough medications. Consequently, neuromodulators are recommended to modulate cough sensitivity [41, 46, 47]. Among the most commonly utilized neuromodulators are gabapentin, baclofen, and pregabalin. Additionally, clinical trials are currently being conducted to address new targets within the cough reflex neural pathway. Among them, P2X3 receptor antagonists show promising applications [59, 60]. In comparison to traditional cough suppressants, targeting CHS has the effect of either minimizing or eliminating pathological cough reflexes while preserving normal protective cough reflexes [38]. The emergence of P2X3 receptor antagonists and other targeted therapies has further strengthened the concept of cough hypersensitivity as a potentially treatable trait in chronic cough. Within a treatable traits framework, identifying and targeting cough hypersensitivity may facilitate a more personalized approach to management beyond conventional etiologic classifications. Future advances in objective biomarkers and precision therapeutics may further improve the identification and treatment of this trait. Importantly, some patients previously classified as having RCC have demonstrated favorable responses to therapies targeting cough hypersensitivity, suggesting that the term “refractory” may not fully reflect the therapeutic potential of this patient population [7].
4. The use of CHS as a diagnostic term serves to facilitate communication with patients in clinical practice [61]. Following a series of diagnostic procedures and empirical treatments, patients diagnosed with UCC/RCC frequently express feelings of anxiety and frustration due to the lack of a definitive diagnosis. From the patient's perspective, terminology itself may influence disease perception and psychological well‐being. Terms such as ICC and UCC emphasize the absence of an identifiable cause, which may be interpreted as diagnostic uncertainty or an incomplete understanding of the disease. Likewise, the term RCC may be perceived as implying treatment failure. These labels may contribute to frustration, anxiety, and reduced confidence in the diagnostic process. The concept of CHS provides a potential mechanism‐based explanation for chronic cough and may help bridge the gap between specific etiologies and chronic cough symptoms [62, 63]. By offering patients a clearer understanding of the mechanisms potentially underlying their symptoms, the use of CHS may facilitate communication between clinicians and patients and improve treatment adherence [61].

The characterization of disease as a diagnostic term, for example, chronic obstructive pulmonary disease (COPD), has been successfully employed in the field of respiratory medicine [20]. As with COPD, the use of CHS as a primary diagnosis does not restrict further investigation of the disease in any respect, and it is of significant value in diagnosis, management, and communication with patients [64]. Furthermore, to illustrate why CHS represents a preferred diagnostic framework over existing terms (ICC, UCC, and RCC), we summarize the respective advantages and limitations of each term in Table 2.

**TABLE 2:** Advantages and limitations of ICC, UCC, RCC, and CHS.

Term	Advantages	Limitations
ICC	Historically significant; emphasizes unknown etiology	Implies diagnostic failure; largely replaced
UCC	Guideline‐endorsed; avoids assumption of no cause	Vague relationship with treatment response
RCC	Clinically relevant for treatment failure	Overlaps with UCC; unclear etiology often ignored
CHS	Pathophysiologically grounded; patient‐aligned; clinician‐friendly; guides therapy	Lacks standardized diagnostic criteria or biomarkers

### 6.2 Current Approaches to the Diagnosis of CHS

Although CHS has gained widespread recognition as a pathophysiological paradigm for chronic cough, the establishment of standardized diagnostic criteria remains an ongoing challenge [46, 65]. Currently, CHS diagnosis relies primarily on clinical recognition rather than a single definitive biomarker or objective test [36]. Recent research has advanced along three complementary assessment domains. First, patient‐reported outcome measures (PROMs) are increasingly utilized to capture hypersensitivity features; validated tools such as the Leicester Cough Questionnaire (LCQ), the newly developed Cough Hypersensitivity Questionnaire (CHQ), and the Cough Hypersensitivity Assessment Test (CHAT) demonstrate moderate‐to‐strong correlations with cough severity, trigger frequency, and quality of life impairment [66, 67, 68]. Second, objective cough provocation tests (e.g., capsaicin or citric acid challenge) remain valuable research instruments for quantifying cough reflex sensitivity, yet their routine clinical application is constrained by methodological heterogeneity, significant inter‐individual variability, and the lack of universally accepted reference thresholds [69, 70]. Third, instrumental assessments such as flexible laryngoscopy and voice evaluation are increasingly explored to identify laryngeal hypersensitivity as a key clinical phenotype of CHS [71]. Consequently, contemporary expert consensus recommends a pragmatic, syndrome‐based approach that integrates characteristic clinical features (e.g., cough triggered by low‐level thermal, mechanical, or chemical stimuli, often accompanied by laryngeal paresthesia), systematic exclusion of competing diagnoses, and targeted therapeutic response, rather than rigid laboratory criteria [46]. Future multidisciplinary standardization of these assessment tools will be essential to harmonize them into a unified diagnostic algorithm [70].

### 6.3 Limitations and Future Perspectives of CHS

Although CHS has provided an important framework for understanding chronic cough, several challenges remain. The diagnosis of cough hypersensitivity is still largely based on clinical symptoms, patient‐reported triggers, and questionnaire‐based assessments, whereas objective biomarkers remain lacking [72]. In addition, chronic cough may represent a panvagal disorder involving both peripheral and central neural pathways, and hypersensitivity arising from different anatomical sites may result in diverse clinical presentations [73]. This may partly explain why typical features of cough hypersensitivity are not observed in all patients with chronic cough or RCC, highlighting the heterogeneity of the disease and the potential existence of different phenotypes and endotypes. Furthermore, therapies targeting cough hypersensitivity pathways are effective in many, but not all, patients, suggesting that additional mechanisms may contribute to cough persistence. Psychological factors may also play a role. Our recent studies demonstrated that somatic symptom disorder (SSD) is common in patients with chronic cough and is associated with poorer treatment response [74, 75].

Nevertheless, these limitations do not diminish the clinical importance of CHS. Although CHS may not fully explain chronic cough in every patient, it remains one of the most well‐supported pathophysiological frameworks for understanding chronic cough and RCC. Future studies focusing on objective biomarkers, disease endotypes, and precision therapeutic approaches may further refine and strengthen the CHS concept.

## 7 Conclusion

The terms UCC and RCC are used to describe etiologic diagnosis and treatment effects, respectively. The relationship between UCC and RCC is complicated by the fact that there is currently no internationally standardized definition of the terms, highlighting the importance of terminology as a topic in this field. Among the conventions proposed, the relationship in which RCC includes UCC is the most recommended and used. The concept of CHS has emerged as an important framework for understanding the pathophysiology of chronic cough. Several authors have proposed that CHS may provide a useful framework for characterizing many patients with chronic cough, particularly those with UCC/RCC. Within this framework, chronic cough associated with known etiologies and UCC/RCC may share common features of cough hypersensitivity. A series of studies have demonstrated the practical value of the CHS concept in clinical evaluation, scientific research, drug development, disease management, and communication with patients. It is of note that the development of tests or biomarkers for the objective diagnosis of cough reflex sensitivity represents an important research goal for future studies.

## Author Contributions

**Yaxing Zhou, Zihang Wang and Yingchun Ren:** provision of study materials, methodology, and writing original draft. **Xianghuai Xu, Li Yu:** conceptualization and validation. **All authors:** manuscript writing and editing. **All authors:** final approval of manuscript.

## Funding

This study was supported by the National Natural Science Foundation of China (No. 82270114 and No. 82570146), the Key Supported Discipline of Health System in Shanghai (2023ZDFC0302), Shanghai Leading Talent Program of Eastern Talent Plan (leading talent program) (BJWS2025069), and the Program of Shanghai Municipal Health Commission Clinical Research (20244Y0147).

## Ethics Statement

The authors have nothing to report.

## Consent

The authors have nothing to report.

## Conflicts of Interest

The authors declare no conflicts of interest.

## Data Availability

The authors have nothing to report.
